# Nonawakening following general anaesthesia after ventriculo-peritoneal shunt surgery: An acute presentation of intracerebral haemorrhage

**DOI:** 10.4103/0019-5049.72650

**Published:** 2010

**Authors:** Achyut Deuri, Devalina Goswami, Mukesh Samplay, Jyotirmoy Das

**Affiliations:** Department of Anaesthesiology, Pt. M.M.M. Hospital, New Delhi, India; 1 Department of Anaesthesiology, D.D.U Hospital, New Delhi, India; 2 Department of Anaesthesiology, Fortis Flt Lt Rajan Dhall Hospital, Vasant Kunj, New Delhi, India

**Keywords:** Intracerebral haemorrhage, non-awakening from anaesthesia, ventriculo-peritoneal shunt

## Abstract

Emergence from general anaesthesia has been a process characterized by large individual variability. Delayed emergence from anaesthesia remains a major cause of concern both for anaesthesiologist and surgeon. The principal factor for delayed awakening from anaesthesia is assumed to be the medications and anaesthetic agents used in the perioperative period. However, sometimes certain non-anaesthetic events may lead to delayed awakening or even non-awakening from general anaesthesia. We report the non-anaesthetic cause (acute intracerebral haemorrhage) for non-awakening following ventriculo-peritoneal shunt surgery.

## INTRODUCTION

Ventriculo-peritoneal shunt (VPS) placement is a routine procedure in daily neurosurgical activity.[1] Although potentially fatal, postoperative intracerebral haematoma has not found its due mention in the literature.[1] Savitz and others reported a 4% incidence of delayed intracerebral haematoma or intraventricular haemorrhage after VPS placement.[2] The intracerebral event can adversely affect the outcome following general anaesthesia in the form of altered sensorium, delayed emergence, or non emergence. In such a situation, the postoperative computerized tomography (CT) scan is the best tool to detect an intracerebral pathology.[2] Further management depends on the location of bleed and other factors.

## CASE REPORT

**Figure 1:**
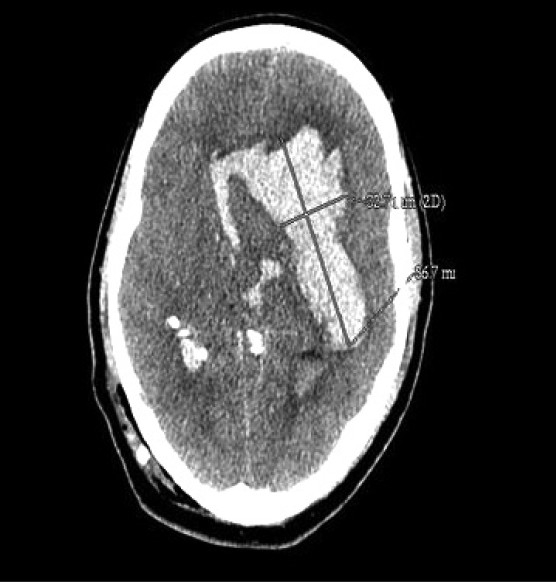
Postoperative CT scan of brain showing massive intracerebral bleed

A 40-year-old 79 kg male presented with the diagnosis of pineal region tumour and hydrocephalus. He had a history of frontal and occipital headache of 1 year duration, gradually decreasing vision and history of one episode of seizure 2 months back. He was on dexamethasone 4 mg 6^th^ hourly and phenytoin sodium 100 mg 8^th^ hourly. On first examination, his blood pressure was 132/80 mmHg, heart rate was 67 beats/min and respiratory rate was 17/min. Preoperative investigations were within normal limits including coagulation profile (Prothrombin time 13.9s with control of 12.5s, international normalized ratio of 1.14, activated partial thromboplastin time of 28s) and platelet count. He was scheduled for a VPS surgery under general anaesthesia. In the operation theatre, his blood pressure was 128/80 mmHg, heart rate 68/min, and Glasgow coma scale (GCS) score was 15/15. He was premedicated with glycopyrrolate 0.2 mg, ondansetron 4 mg and midazolam 1 mg. Anaesthesia was induced with thiopentone sodium 250 mg and fentanyl 150 *μ*g. Vecuronium 8 mg was used to facilitate tracheal intubation. Anaesthesia was maintained with nitrous oxide and isoflurane 1% to 1.2% concentration with an inspired fraction of oxygen (FiO_2_) of 0.4. The surgeon inserted the ventricular end of the shunt in third attempt. Non-haemorrhagic cerebrospinal fluid was drained under high pressure. Rest of the surgical procedure was uneventful. Throughout the operative period, patient remained haemodynamically stable. Residual neuromuscular blockade was reversed with neostigmine 3.0 mg and glycopyrrolate 0.4 mg. The respiratory parameters and the core temperature were within normal limits and pupils were reactive and normal in size. However, the patient remained unconscious even after 10 min with only localizing response to painful stimuli, hence the patient was shifted to intensive care unit (ICU). In the ICU, he was stable with blood pressure of 126/78 mmHg and heart rate of 77/min. Arterial gases and glucose were within normal limits. One hour after the surgery, pupils were dilated bilaterally and sluggishly reacting. Therefore, 200 ml of mannitol 20% and furosemide 40 mg were administered. CT scan of brain showed massive intracerebral bleed with bleeding into the left basal ganglia, brain stem and intraventricular area [Figure 1]. The patient remained unconscious and died on the second postoperative day due to this intracranial catastrophe

## DISCUSSION

Early emergence from anaesthesia is essential following neurosurgery for neurological evaluation. Zelcer and Wells[3] found a 9% incidence of unresponsiveness at the end of 15 min after general anaesthesia among 443 mixed surgical patients. Arousal beyond 15 min[3] and 30 min[4] is labelled as delayed emergence. Residual anaesthesia may either give the false impression of a neurological deficit[5] or prevent the early diagnosis of a developing intracranial lesion like haematoma, herniation and cerebral infarction. A patient with altered sensorium is also at greater risk for airway obstruction, hypoxaemia, hypercarbia and aspiration.[6]

The most common cause for delayed awakening following anaesthesia is medications and anaesthetic agents used in the perioperative period.[7,8] There may be an overdose (absolute or relative in susceptible patients) of medications. Emergence from anaesthetic agents depend on the tissue uptake of the drug, average concentration used and the duration of exposure.[9] Certain underlying metabolic disorders such as hypoglycaemia, severe hyperglycaemia, electrolyte imbalance (especially hyponatraemia), hypoxia, hypercapnia, central anticholinergic syndrome, chronic hypertension, liver disease, hypoalbuminemia, uraemia and severe hypothyroidism may also be responsible for delayed recovery after anaesthesia.[9] Preoperative medications such as opioids and sedatives and hypothermia can further interfere with postoperative recovery.

Intraoperative cerebral hypoxia, haemorrhage, embolism or thrombosis also can manifest as delayed awakening from anaesthesia.[10] Although pupil size is not always a reliable indicator of central nervous system integrity, a fixed and dilated pupil in the absence of anticholinergic medication or ganglionic blockade may be an ominous sign. Therefore, patients with delayed emergence from anaesthesia after intracranial surgery undergo emergency CT scan or cerebral angiography.[9]

Small haemorrhages into the ventricles, subependymal area and around the ventricular catheters are frequently seen following VPS surgery.[11] Udvarhelyi and others,[12] first reported two cases with intracerebral haemorrhage after VPS insertion. The possible mechanisms of intracerebral haemorrhages after shunt insertion include a bleeding disorder, anticoagulant therapy, surgery induced disseminated intravascular coagulation, disruption of an intracerebral vessel by the catheter, haemorrhage into an intracerebral tumour, multiple attempts at localizing the ventricles, haemorrhage from a vascular malformation or spontaneous vascular rupture secondary to progressive degenerative vascular changes.[2,11,12] Bleeding secondary to ventricular cannulation may be readily detected on intraoperative ultrasonography and postoperative CT or magnetic resonance imaging studies. Performing an urgent CT scan following non-emergence from general anaesthesia in patient requiring repeated attempts at ventricular catheter insertion is highly desirable to exclude any intracranial event and address treatable cause.[2]

## CONCLUSION

Early emergence from anaesthesia is highly desirable following neurosurgical procedures. Delayed emergence, often blamed on the anaesthetic agents may not always be the culprit as seen in the current report. When other causes are excluded the possibility of an acute intracranial event as an aetiology for delayed awakening should be strongly considered.
